# Individualizing the lifesaving journey for calciphylaxis: addressing rapidly progressive attacks with multidimensional and AI research for regenerative medicine

**DOI:** 10.1080/0886022X.2024.2392846

**Published:** 2024-09-05

**Authors:** Ningning Wang, Andrea Angioi, Nicolas Hanset, Xiaoxue Ye, Shijiu Lu, Yi Zhu

**Affiliations:** a Department of Nephrology, the First Affiliated Hospital of Nanjing Medical University, Jiangsu Province Hospital, Nanjing, Jiangsu, China; b Department of Nephrology, Azienda Ospedaliera G. Brotzu, Cagliari, Italy; c Department of Nephrology, Tenon Hospital, Paris, France; d Putuo District Central Hospital of Shanghai, Shanghai, China; e School of Medicine, Westlake University, Hangzhou, Zhejiang, China; f Westlake Center for Intelligent Proteomics, Westlake Laboratory of Life Sciences and Biomedicine, Hangzhou, Zhejiang, China; g Research Center for Industries of the Future, School of Life Sciences, Westlake University, Hangzhou, Zhejiang, China

## The challenges of calciphylaxis prompted the development of this article collection

Calciphylaxis is a challenging orphan disease (ORPHA:280062). The painful and ischemic cutaneous lesions mainly affect patients with chronic kidney disease (CKD), also known as calcific uremic arteriolopathy (CUA) [1,2]. It presents as progressive and non-healing skin lesions that are histologically characterized by calcification, fibrointimal hyperplasia, and thrombosis of microvessels [2]. Due to the heterogeneity of clinical manifestations, skin biopsy is considered the gold standard diagnostic method; however, it is an invasive procedure [3].

In 1961, Selye first proposed the concept of “calciphylaxis”, which described the presence of calcium deposition in skin tissue of uremic rats after stimulation by sensitizing agents, without observing skin ischemic necrosis [4]. However, calciphylaxis in humans is not an inducible hypersensitivity reaction disease. The scarcity of animal models that closely mimic the clinical presentation hampers an in-depth understanding of the pathogenesis and restricts the scope of translational research for calciphylaxis [5,6].

Currently, the existing literature on calciphylaxis primarily consists of retrospective studies and case series, lacking well-defined guidelines. For primary care physicians, especially those not specialized in nephrology, education on calciphylaxis prevention knowledge and the identification of early clinical manifestations should be strengthened. The multidisciplinary targeted approach, involving the nephrology department, dermatology department, pathology department, hematology department, burn and plastic surgery department, infection department, pain management department, and so on, is considered to be the primary method of treatment. Until now, there has been no proven therapy for calciphylaxis, with the median survival time is three months and a 1-year mortality rate of up to 80% due to ulceration and infection [5,6].

Given the sense of powerlessness experienced by patients, their families, and medical personnel in relation to calciphylaxis, there is an urgent need to explore new avenues in the research of its mechanisms, precise diagnostic and therapeutic measures. Chronic kidney disease-mineral and bone disorder (CKD-MBD), especially vascular calcification (VC), shares certain similarities with calciphylaxis in terms of risk factors, clinical manifestations, and treatment methods. In light of this, we have compiled an article collection (AC) titled “Calciphylaxis and CKD-MBD: Ongoing Challenges and Opportunities”. Seventeen manuscripts have been included, with 5 focused on calciphylaxis and 12 on CKD-MBD. The authors come from China, Canada, and Germany.

## Multilayered contributions about calciphylaxis within article collection

At present, there is no accepted diagnostic standard for calciphylaxis. Dr Yuqiu Liu et al. from China have proposed a modified approach for identifying calciphylaxis at early stages. They emphasize the significance of identifying atypical skin lesions, utilizing advanced radiological technology, and promptly performing skin biopsy [7]. Of note, non-uremic calciphylaxis (NUC) is frequently misdiagnosed. Sabine Yousuf et al. from Germany conducted a retrospective review of 60 patients with calciphylaxis, including 21 patients with NUC. They identified significant differences in comorbidities and treatment modalities, and underscoring the need for distinct management strategies for each group. The predilection sites of skin lesions were the lower legs in 88%. They emphasize the role of hypercoagulability, CKD-MBD, abnormal lipid metabolism, malnutrition, and loop diuretics in the occurrence of calciphylaxis [8].

A female patient with calciphylaxis, who underwent mechanical cardiac valve replacement and was being treated with warfarin, passed away due to septic shock. The patient received symptomatic treatments, including anticoagulation and sodium thiosulfate (STS). Shichu Liang and colleagues emphasized the need for alternative anticoagulant strategies due to the risks associated with warfarin [9]. Warfarin is a vitamin K antagonist (VKA) that inhibits the vitamin K-dependent γ-glutamyl carboxylation of proteins, including osteocalcin and matrix Gla proteins. This, in turn, increases the risk of osteoporotic fractures and vascular calcification [10]. A study showed that about 50% of CUA patients in a German registry used VKAs [11]. Clinically, warfarin-associated calciphylaxis usually occurs below the knee and manifests as sclerotic plaques with overlying reticulopenic purpura and central necrosis, with pathological features including vascular calcification, ischemia, necrosis, and thrombosis, among others [12]. Chloé Lajoie and the team conducted a study in Canada which reported on 12 patients with calciphylaxis. The patients presented with comorbidities such as diabetes, use of calcium-based phosphate binders and intravenous iron. They adopted a multimodality approach, including STS, to treat calciphylaxis in ESKD patients. The authors introduced the average duration, cumulative dose, positive response rate, and complete response rate of patients treated with STS. The one-year mortality rate was reported as 25% [13]. These therapy experiences involving STS hold significant reference value.

Dr. Anning Bian et al. from China reported a case of uremic calciphylaxis who received a salvage strategy involving the application of intravenous and local human amniotic-derived mesenchymal stem cells (hAMSCs) therapy. Identification of coagulation profile, including both congenital and acquired thrombophilias are proposed to be assessed for the new onset calciphylaxis patients. Hypercoagulability is expected to be improved through the use of novel hAMSC treatment [14].

## Themes overview of CKD-MBD within article collection

In this AC, a series of studies have yielded critical findings regarding CKD-MBD, cardiovascular events, and therapeutic strategies. Li Sun et al. from China revealed MBD persisted after kidney transplant (KT), showing a close relationship with hyperparathyroidism, high bone turnover, and glucocorticoid accumulation [15]. They further demonstrated that vascular calcification progressed, while left ventricular remodeling partially improved after KT [16, 17]. Active control of risk factors such as MBD and hyperglycemia; shortened the waiting time before KT will improve the survival rate of KTRs. Iguratimod, a drug promoting bone formation and inhibiting bone resorption in rheumatoid arthritis patients, can reduce bone turnover markers (BTMs) and may attenuate vascular calcification in the first year after KT, suggesting its safety and potential therapeutic value [18].

Dr Xinfang Tang et al. validated a nomogram model based on clinical risk factors to predict the risk of severe coronary calcification in ESKD patients. Dr Chaoying Kuang et al. have proposed that the risk factors for osteoporosis differ according to gender and renal function stages in pre-dialysis CKD patients. In order to facilitate the rapid screening of patients at high risk of osteoporosis, the authors constructed clinical prediction models in the form of nomograms [19]. These predictive models have the potential to significantly decrease the incidence of cardiovascular and fracture events, as well as reduce mortality rates, especially among CKD-MBD patients in primary healthcare settings [20].

Ying Gong et al. proposed that metastasis-associated lung adenocarcinoma transcript 1 (MALAT1) may serve as a potential therapeutic target for vascular calcification related diseases. This effect is partially achieved through mediating the microRNAs-30c/Runx2 axis [21]. Qiaojing Liang proposed that lower *miR-16-5p* expression levels is a potential risk factor of vascular calcification in maintenance hemodialysis patients [22]. Rao Fu et al. reported that bone marrow (BM)-derived macrophages (BMMs) from renal osteodystrophy (ROD) rats displayed overproduction of proinflammatory cytokines and increased osteoclast differentiation, accompanied by nuclear factor κB (NF-κB) signaling activation. Exosomes derived from ROD (ROD-Exos) activate NF-κB signaling. The roles of differentially expressed miRNAs, particularly miR-9a-5p, need to be further clarified in the pathogenesis of high-turnover ROD [23].

Yilin Wang et al. emphasized that adequate intake of vitamin D and control of tartrate-resistant acid phosphatase 5b (TRACP-5b) levels will help reduce the occurrence and progression of osteopenia/sarcopenia in HD patients [24]. Fan Li et al. investigated the relationships between blood bone metabolic biomarkers and anemia in CKD patients, revealing that adjusted calcium, phosphorus, iPTH, and α-klotho levels were correlated with hemoglobin levels in CKD patients. The study suggested that correction of bone metabolic disorders may be a therapeutic strategy for anemia treatment [25]. A meta-analysis examined the efficacy and safety of sevelamer as a treatment for hyperphosphatemia in hemodialysis patients, which included data from 34 randomized controlled trials (RCTs). This study was conducted by Qian Zeng et al. [26]. They indicated that sevelamer could be beneficial in preventing all-cause mortality and vessel calcification among CKD patients, and induced less hypercalcemia and hyperphosphatemia when compared with calcium-based binders in CKD5D individuals. In addition, studies have shown that hyperphosphatemia is one of the independent risk factors for patients with CUA [27, 28], and that lowering blood phosphorus levels may provide benefits to patients with CUA.

## Insights from CKD-MBD illuminate the investigation of calciphylaxis

The manuscripts related to calciphylaxis in this article collection have filled important gaps in comparative clinical and epidemiological studies. In order to enhance our understanding of the mechanisms underlying calciphylaxis, it is advisable to gain insights from CKD-MBD, a condition that has been frequently demonstrated to coexist with calciphylaxis.

This article collection highlights several important factors in the development of vascular calcification, including inflammation, drug side effects (e.g. glucocorticoids), and the significance of the local microenvironment. Additionally, the interconnectedness among metabolism disorders, vascular injuries, renal osteodystrophy, and various promising intervention methods is further elucidated. These key findings provide valuable research insights for risk stratification, comprehensive systemic assessment, and treatment strategies for patients with calciphylaxis.

However, vascular calcification is a frequently observed complication in patients with CKD, making it challenging to elucidate the mechanisms that contribute to the rapid progression of calciphylaxis and its associated high mortality rate. A recent meta-analysis also reported no significant association between the administration of intravenous STS and improvements in skin lesions or survival benefits in CUA patients [29].

## Rapidly progressing vascular injury in the lifesaving journey of calciphylaxis

Exploration is warranted to investigate the differences in the pathogenesis of vascular injury between patients with calciphylaxis and those with vascular calcification in CKD-MBD. Further investigation is essential to understanding the causal relationships between the harmful acute impacts, inducing ischemia and inflammatory responses, on rapidly progressive microvascular injuries in the calciphylaxis patients.

Noninvasive diagnosis and prognostic biomarkers for calciphylaxis, the identification of pivotal risk factors for NUC and CUA, and the multimodal discrimination diagnostic tool are highly needed. Addressing rapidly progressing vascular injury of calciphylaxis is challenging through the investigation of a single drug using traditional clinical designs. Considering the specific clinical scenarios in detail, the inclusion of novel and diverse research methods provides significant opportunities for studying this devastating disease.

## Partnering with patients and families for the research agenda of calciphylaxis

Because of the trust, dedication and persistence exhibited by rare disease patients and their families, we should encourage them to participate as research partners and collaborate closely with clinical physicians and scientists. Actively listening to the voices of patients and their families will undoubtedly shed light on our understanding of early diagnosis, disease progression, treatment response, and person-centered research directions for calciphylaxis. A 2020 survey on rare disease in China, which included both children and adult patients, demonstrated an average delay in diagnosis of 1.4 years and an average of 3.2 hospitals visited prior to diagnosis [30, 31]. Currently, there is a lack of relevant information regarding calciphylaxis.

We recommend establishing multicenter registries and collaborative efforts in order to figure out the clues of acute impacts for this orphan disease. After diagnosis, patients and their families contribute to the efforts to trace the progressive development of calciphylaxis, providing valuable insights based on their firsthand experiences. This helps medical teams expand their interdisciplinary expertise concerning clinical details and risk factors from a more systematic perspective in the initial stage. It also helps them build predictive models, aiding in early diagnosis and precise treatment [32].

Patients and their families, with self-management skills, a willingness to continue learning, and a positive and cooperative attitude, will become essential members to inform the research agenda. By familiarizing themselves with the atypical clinical manifestations of calciphylaxis, adhering to appropriate daily wound care practices, and understanding the necessary dietary and medication precautions, patients and their families can contribute to the early diagnosis and effective management of their skin wounds. Furthermore, they can join as members to explore innovative therapeutic strategies and ultimately improve the outcomes related to calciphylaxis. Trust is a fundamental component of the doctor-patient relationship [33]. They will provide valuable feedback on patients’ needs and priorities, personal data, share their experiences with the disease, their understanding of research, and play a vital role in decision-making and promoting the dissemination of medical knowledge. This will ultimately enhance the comprehensiveness, reliability, and feasibility of clinical research while also contributing to reducing costs. Additionally, they will serve as role models and sources of inspiration for other patients.

## Building multidimensional research system based on specific molecular makeup

The principles of molecular biology suggest that collectives along the axis of the central dogma capture the functional and biochemical state of the cells. It is well known that the translation of genomic information into protein function is tightly controlled at multiple levels. Briefly, the central dogma of biology explains how the information contained in the genome is converted into an ensemble of proteins. Additionally, certain genotypic variability will alter the structure and function of a protein module and result in a disease phenotype. Furthermore, the biochemical functions of a cell are catalyzed by macromolecular modules consisting of multiple subunits. These molecular characteristics could be used as biomarkers to diagnose the disease and predict its course and treatment.

Precision medicine attempts to select the optimal therapy for individual patients based on their specific molecular makeup. Technological advances in multi-omics have increased the feasibility of quantifying molecular variability and identifying disease-specific deviations. Mass spectrometry-based bottom-up proteomics, including data-dependent acquisition (DDA, shotgun) proteomics, data-independent acquisition (DIA) proteomics, and targeted proteomics, have widely been used in large-scale clinical samples for biomarker discovery and validation [34–36]. Disease stratification and therapy selection, which are based on the analysis of large-scale molecular data that encompass comprehensive information about disease pathogenesis, have been demonstrated to be both feasible and effective in a wide range of diseases, including cancer and CKD.

The multidimensional and artificial intelligence (AI) technologies for multiple organs, with small samples and minimal amounts of tissue, have made it possible to conduct comprehensive analysis and tailored treatment plans for complex diseases. This exploration paradigm will provide valuable knowledge about the mechanisms and clinical translational research of not only calciphylaxis, but also other emergency, critical, and orphan diseases.

**Figure 1.**
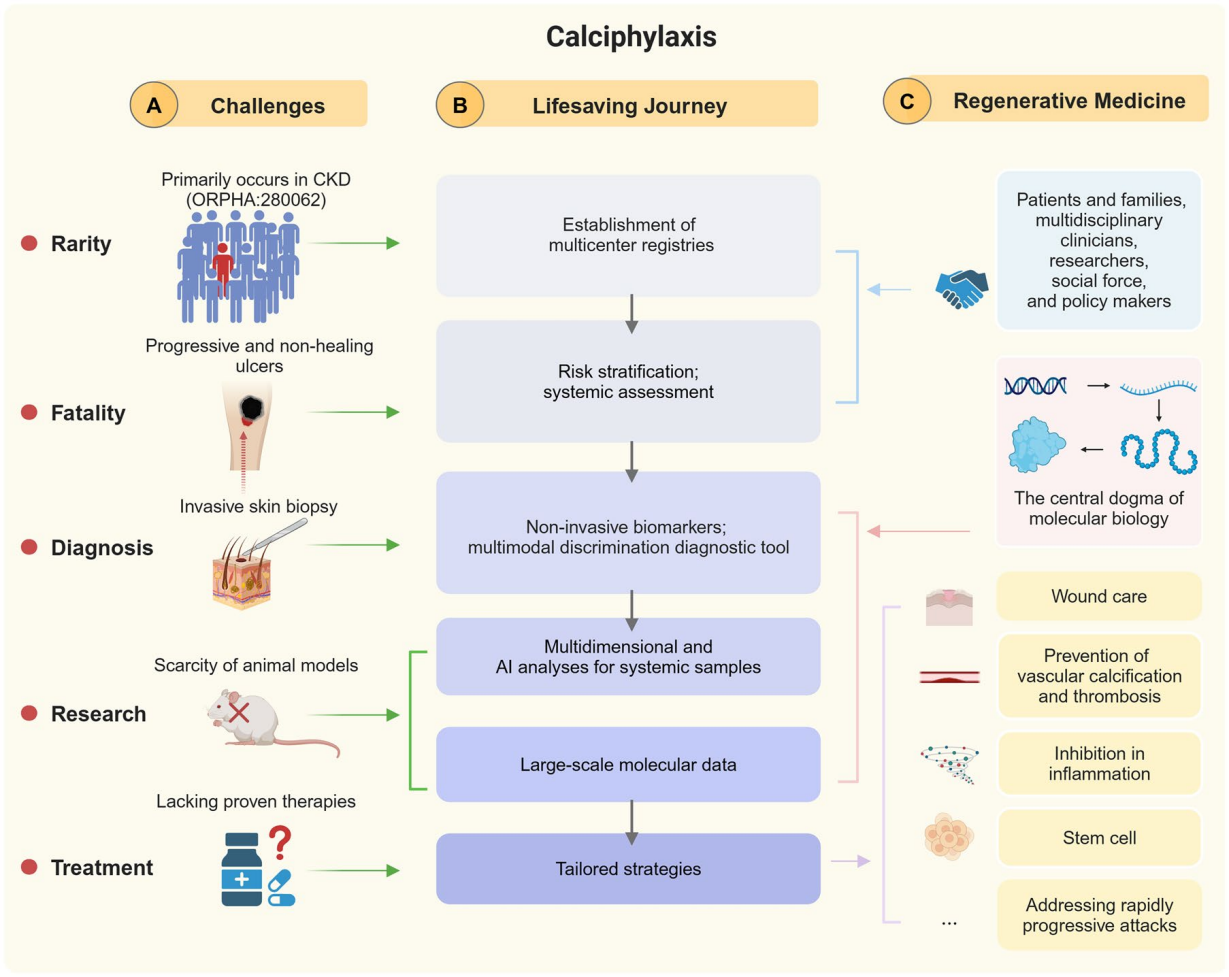
Lifesaving journey of calciphylaxis to regenerative medicine with multidimensional and AI research.

The diagram illustrates the lifesaving journey for calciphylaxis to regenerative medicine, incorporating multidimensional and AI research, as presented in Figure 1.

## Conclusion

Insights from CKD-MBD have provided valuable clues for research on calciphylaxis. It is essential to prioritize the investigation of rapidly progressive attacks, particularly ischemia and inflammation, for this orphan but challenging disease. As crucial research partners, the trust, determination, and resilience of patients and their families are of utmost importance. Embracing new technologies across multiple disciplines, constructing noninvasive diagnostic and early prediction models, identifying treatment targets through multidimensional research, and promoting regenerative medicine under the guidance of biomarkers are the North Star in the person-centered lifesaving journey for calciphylaxis.

## References

[1] Nigwekar SU, Thadhani R, Brandenburg VM. Calciphylaxis. N Engl J Med. 2018;378(18):1704–1714. doi: 10.1056/NEJMra1505292. 29719190

[2] Rick J, Strowd L, Pasieka HB, et al. Calciphylaxis: part I. Diagnosis and pathology. J Am Acad Dermatol. 2022;86(5):973–982. doi: 10.1016/j.jaad.2021.10.064. 35114300

[3] Rotondi S, De Martini N, Tartaglione L, et al. On the role of skin biopsy in the diagnosis of calcific uremic arteriolopathy: a case-based discussion. J Nephrol. 2020;33(4):859–865. doi: 10.1007/s40620-019-00678-z. 31792896

[4] Selye H, Gentile G, Prioreschi P. Cutaneous molt induced by calciphylaxis in the rat. Science. 1961;134(3493):1876–1877. doi: 10.1126/science.134.3493.1876. 13910515

[5] Gallo Marin B, Aghagoli G, Hu SL, et al. Calciphylaxis and kidney disease: a review. Am J Kidney Dis. 2023;81(2):232–239. doi: 10.1053/j.ajkd.2022.06.011. 35970430

[6] Nigwekar SU, Kroshinsky D, Nazarian RM, et al. Calciphylaxis: risk factors, diagnosis, and treatment. Am J Kidney Dis. 2015;66(1):133–146. doi: 10.1053/j.ajkd.2015.01.034. 25960299 PMC4696752

[7] Liu Y, Zhang X. Early diagnosis strategy of calciphylaxis in dialysis patients. Ren Fail. 2023;45(2):2264407. doi: 10.1080/0886022X.2023.2264407. 37795796 PMC10557543

[8] Yousuf S, Busch D, Renner R, et al. Clinical characteristics and treatment modalities in uremic and non uremic calciphylaxis - a dermatological single-center experience. Ren Fail. 2024;46(1):2297566. doi: 10.1080/0886022X.2023.2297566. 38178572 PMC10773653

[9] Liang S, Guan M, Liu Z, et al. Sailing between scylla and charybdis-anticoagulation dilemma in a patient with calciphylaxis and mechanical cardiac valve replacement: a case report and literature review. Ren Fail. 2023;45(2):2264401. doi: 10.1080/0886022X.2023.2264401. 37799073 PMC10561572

[10] Yamagishi SI. Concerns about clinical efficacy and safety of warfarin in diabetic patients with atrial fibrillation. Cardiovasc Diabetol. 2019;18(1):12. doi: 10.1186/s12933-019-0818-0. 30691466 PMC6348611

[11] Brandenburg VM, Kramann R, Rothe H, et al. Calcific uraemic arteriolopathy (calciphylaxis): data from a large nationwide registry. Nephrol Dial Transplant. 2017;32(1):126–132. doi: 10.1093/ndt/gfv438. 26908770

[12] Yu WY, Bhutani T, Kornik R, et al. Warfarin-associated nonuremic calciphylaxis. JAMA Dermatol. 2017;153(3):309–314. doi: 10.1001/jamadermatol.2016.4821. 28099971 PMC5703198

[13] Lajoie C, Ghanemi A, Bourbeau K, et al. Multimodality approach to treat calciphylaxis in end-stage kidney disease patients. Ren Fail. 2023;45(2):2256413. doi: 10.1080/0886022X.2023.2256413. 37724534 PMC10512890

[14] Bian A, Ye X, Wang J, et al. Therapeutic effects and mechanism of human amnion-derived mesenchymal stem cells on hypercoagulability in a uremic calciphylaxis patient. Ren Fail. 2023;45(1):2218483. doi: 10.1080/0886022X.2023.2218483. 37293809 PMC10259294

[15] Sun L, Wang Z, Zheng M, et al. Mineral and bone disorder after kidney transplantation: a single-center cohort study. Ren Fail. 2023;45(1):2210231. doi: 10.1080/0886022X.2023.2210231. 37183797 PMC10187110

[16] Sun L, Huang Z, Fei S, et al. Vascular calcification progression and its association with mineral and bone disorder in kidney transplant recipients. Ren Fail. 2023;45(2):2276382. doi: 10.1080/0886022X.2023.2276382. 37936391 PMC10653689

[17] Sun L, Zhang D, Liu J, et al. Left ventricular remodeling and its association with mineral and bone disorder in kidney transplant recipients. Ren Fail. 2024;46(1):2300303. doi: 10.1080/0886022X.2023.2300303. 38263697 PMC10810624

[18] Sun L, Tao J, Han Z, et al. Efficacy of iguratimod on mineral and bone disorders after kidney transplantation: a preliminary study. Ren Fail. 2023;45(2):2256418. doi: 10.1080/0886022X.2023.2256418. 37905940 PMC11001337

[19] Kuang C, Shang J, Ma M, et al. Risk factors and clinical prediction models for osteoporosis in pre-dialysis chronic kidney disease patients. Ren Fail. 2024;46(2):2361802. doi: 10.1080/0886022X.2024.2361802. 38874080 PMC11182074

[20] Tang X, Qian H, Lu S, et al. Predictive nomogram model for severe coronary artery calcification in end-stage kidney disease patients. Ren Fail. 2024;46(2):2365393. doi: 10.1080/0886022X.2024.2365393. 38874139 PMC11232636

[21] Gong Y, Zhong Q, Xia Y, et al. Long non-coding RNA MALAT1 sponges miR-30c to promote the calcification of human vascular smooth muscle cells by regulating Runx2. Ren Fail. 2023;45(1):2204953. doi: 10.1080/0886022X.2023.2204953. 37125614 PMC10134953

[22] Liang Q, Fu C, Liu Y, et al. Association of plasma microRNA-16-5p and abdominal aortic calcification in maintenance hemodialysis patients. Ren Fail. 2024;46(2):2368091. doi: 10.1080/0886022X.2024.2368091. 39049724 PMC11275526

[23] Fu R, Meng K, Zhang R, et al. Bone marrow-derived exosomes promote inflammation and osteoclast differentiation in high-turnover renal osteodystrophy. Ren Fail. 2023;45(2):2264396. doi: 10.1080/0886022X.2023.2264396. 37870853 PMC11001343

[24] Wang Y, Ma W, Pu J, et al. Interrelationships between sarcopenia, bone turnover markers and low bone mineral density in patients on hemodialysis. Ren Fail. 2023;45(1):2200846. doi: 10.1080/0886022X.2023.2200846. 37122165 PMC10134952

[25] Li F, Ye X, Yang G, et al. Relationships between blood bone metabolic biomarkers and anemia in patients with chronic kidney disease. Ren Fail. 2023;45(1):2210227. doi: 10.1080/0886022X.2023.2210227. 37170583 PMC10184590

[26] Zeng Q, Zhong Y, Yu X. Meta-analysis of the efficacy and safety of sevelamer as hyperphosphatemia therapy for hemodialysis patients. Ren Fail. 2023;45(1):2210230. doi: 10.1080/0886022X.2023.2210230. 37272189 PMC10243412

[27] Mazhar AR, Johnson RJ, Gillen D, et al. Risk factors and mortality associated with calciphylaxis in end-stage renal disease. Kidney Int. 2001;60(1):324–332. doi: 10.1046/j.1523-1755.2001.00803.x. 11422768

[28] Liu Y, Zhang X, Xie X, et al. Risk factors for calciphylaxis in Chinese hemodialysis patients: a matched case-control study. Ren Fail. 2021;43(1):406–416. doi: 10.1080/0886022X.2021.1884094. 33641601 PMC7927988

[29] Wen W, Portales-Castillo I, Seethapathy R, et al. Intravenous sodium thiosulphate for calciphylaxis of chronic kidney disease: A systematic review and meta-analysis. JAMA Netw Open. 2023;6(4):e2310068. doi: 10.1001/jamanetworkopen.2023.10068. 37099293 PMC10134003

[30] Li X, Liu M, Lin J, et al. A questionnaire-based study to comprehensively assess the status quo of rare disease patients and care-givers in China. Orphanet J Rare Dis. 2021;16(1):327. doi: 10.1186/s13023-021-01954-7. 34294091 PMC8296703

[31] Xie J, Zhang S. A patient-centric, coordinated care model for rare diseases: The multidisci-plinary consultation program at Peking Union Medical College Hospital. J. Rare Dis. 2024;3(1):77–78. doi: 10.12376/j.issn.2097-0501.2024.01.010.

[32] Harrison TG, Elliott MJ, Tonelli M. Integrating the patient voice: patient-centred and equitable clinical risk ­prediction for kidney health and disease. Curr Opin Nephrol Hypertens. 2024;33(4):456–463. doi: 10.1097/MNH.0000000000000993. 38656234

[33] Greene J, Wolfson D. Physician perspectives on building trust with patients. Hastings Cent Rep. 2023;53 Suppl 2(Suppl 2):S86–S90. doi: 10.1002/hast.1528. 37963052

[34] Nie X, Qian L, Sun R, et al. Multi-organ proteomic landscape of COVID-19 autopsies. Cell. 2021;184(3):775–791.e14. doi: 10.1016/j.cell.2021.01.004. 33503446 PMC7794601

[35] Cai X, Xue Z, Zeng FF, et al. Population serum proteomics uncovers a prognostic protein classifier for metabolic syndrome. Cell Rep Med. 2023;4(9):101172. doi: 10.1016/j.xcrm.2023.101172. 37652016 PMC10518601

[36] Wang Z, Wang H, Zhou Y, et al. An individualized protein-based prognostic model to stratify pediatric patients with papillary thyroid carcinoma. Nat Commun. 2024;15(1):3560. doi: 10.1038/s41467-024-47926-w. 38671151 PMC11053152

